# In Vitro and In Vivo Antioxidant and Anti-Hyperglycemic Activities of Moroccan Oat Cultivars

**DOI:** 10.3390/antiox6040102

**Published:** 2017-12-06

**Authors:** Ilias Marmouzi, El Mostafa Karym, Nezha Saidi, Bouchra Meddah, Mourad Kharbach, Azlarab Masrar, Mounya Bouabdellah, Layachi Chabraoui, Khalid El Allali, Yahia Cherrah, My El Abbes Faouzi

**Affiliations:** 1 Laboratoire de Pharmacologie et Toxicologie, équipe de Pharmacocinétique, Faculté de Médicine et Pharmacie, University Mohammed V in Rabat, BP 6203, Rabat Instituts, Rabat 10100, Morocco; b.meddah@um5s.net.ma (B.M.); cherrahy@yahoo.fr (Y.C.); myafaouzi@yahoo.fr (M.E.A.F.); 2 Laboratoire de Biochimie et Neurosciences, FST, Université Hassan I, BP 577, Settat 26000, Morocco; karimex100@hotmail.com; 3 Regional Office of Rabat, National Institute for Agricultural Research, P.O. Box 6570, Rabat Institutes, Rabat 10101, Morocco; nezsaidi@yahoo.fr; 4 Pharmaceutical and Toxicological Analysis Research Team, Laboratory of Pharmacology and Toxicology, Faculty of Medicine and Pharmacy, University Mohammed V, Rabat 10100, Morocco; mourad.kharbach@hotmail.fr; 5 Department of Analytical Chemistry, Applied Chemometrics and Molecular Modelling, CePhaR, Vrije Universiteit Brussel (VUB), Laarbeeklaan 103, B-1090 Brussels, Belgium; 6 Central Laboratory of Biochemistry, Ibn Sina Hospital, Rabat 10100, Morocco; amasrar@yahoo.fr (A.M.); monybouabdellah@yahoo.com (M.B.); lchabraoui@yahoo.fr (L.C.); 7 Comparative Anatomy Unit-URAC-49, Hassan II Agronomy and Veterinary Institute, Rabat 10101, Morocco; khalid_elallali@yahoo.fr

**Keywords:** streptozotocin-nicotinamide, anti-hyperglycemic, hybrid Oat, digestive enzyme

## Abstract

Improvement of oat lines via introgression is an important process for food biochemical functionality. This work aims to evaluate the protective effect of phenolic compounds from hybrid Oat line (F11-5) and its parent (Amlal) on hyperglycemia-induced oxidative stress and to establish the possible mechanisms of antidiabetic activity by digestive enzyme inhibition. Eight phenolic acids were quantified in our samples including ferulic, *p*-hydroxybenzoic, caffeic, salicylic, syringic, sinapic, *p*-coumaric and chlorogenic acids. The Oat extract (2000 mg/kg) ameliorated the glucose tolerance, decreased Fasting Blood Glucose (FBG) and oxidative stress markers, including Superoxide dismutase (SOD), Catalase (CAT), Glutathione peroxidase (GPx), Glutathione (GSH) and Malondialdehyde (MDA) in rat liver and kidney. Furthermore, Metformin and Oat intake prevented anxiety, hypercholesterolemia and atherosclerosis in diabetic rats. In vivo anti-hyperglycemic effect of Oat extracts has been confirmed by their inhibitory activities on α-amylase (723.91 μg/mL and 1027.14 μg/mL) and α-glucosidase (1548.12 μg/mL & 1803.52 μg/mL) enzymes by mean of a mixed inhibition.

## 1. Introduction

Oxidative stress (OS) has been implicated as a contributor to both the onset and progression of diabetes [1]. In physiologic concentrations, endogenous reactive oxygen species (ROS) help to maintain homeostasis. However, when ROS accumulate in excess for prolonged periods of time, they cause chronic oxidative stress and adverse effects [2]. The mitochondrial overproduction of ROS in hyperglycemia has been postulated to cause redox imbalance, oxidative insults, mitochondrial dysfunction, and cell death. The overwhelmed free radicals can damage DNA integrity, membrane lipids and protein function by oxidation and lead to functional abnormalities, apoptosis or necrosis [3]. Generally, many of the common risk factors, such as obesity, increased age, and unhealthy eating habits, enhance pro-oxidative milieu, and may contribute to the development of insulin resistance [4,5], β-cell dysfunction, impaired glucose tolerance, and mitochondrial dysfunction [1], which can ultimately lead to the diabetic disease state. Data from experimental and clinical studies suggest an inverse association between insulin sensitivity and radical oxygenated species (ROS) levels [6]. OS also contributes in the development of diabetic complications, including diabetic retinopathy, nephropathy, peripheral neuropathy, and cardiovascular disease [7].

As can be expected, nutritional therapies that alter or disrupt OS mechanisms may serve to reduce the risk of development of diabetes [1]. Especially, cereal foods such as oats are recommended for healthy diets as being recognized sources of antioxidants [8], contributing to the management of the oxidative stress and consequently the amelioration of the diabetic status [9]. Above all, the phenolic content in Oat reveals that it may serve as an excellent dietary source of natural antioxidants [10]. Antioxidant therapy can protect pancreatic cells from apoptosis and preserve their functions [11]. Therefore, the higher antioxidant effects a compound might have, the higher the positive effects in diabetes prevention. Many previous works has reported anti-hyperglycemic effect of whole Oat products and β-glucans [12,13]. However few papers described the protective effect of Oat phenolics against oxidative damage in diabetic and cellular models and simultaneously evaluated the cognitive impairments that result from diabetic and oxidative environment. Accordingly, diabetes has been shown to be strongly implicated in Alzheimer, memory loss, depression and anxiety [14,15]. Thus, there is a need to evaluate the protective effect of nutritional intervention against cognitive diabetic complications. Moreover, it is established that Oat mechanisms of antidiabetic activity are diverse and complementary; however Oat interactions with digestive enzymes are not very well described, especially α-amylase and α-glucosidase [16,17], unless recently for some disaccharides [18].

Nowadays, the food industry is more and more directed to minor crops that have been underestimated and mainly used in animal feeding in the past decades [10,19]. In this regard, it has been shown that improvement of oat lines via introgression from tetraploids has a promising effect on Oat nutritional characteristics [20]. In our previous investigation [10], we have described the nutritional characteristics of Moroccan Oat varieties; in this focus the current work aims to compare the therapeutic and preventive effect of a Moroccan hybrid Oat (F11-5) and its parent (Amlal), on oxidative damage and antioxidants enzymes under diabetic status, also to evaluate their antioxidant effects in *Tetrahymena* model, and to characterize the inhibitory properties on digestive enzymes (α-amylase and α-glucosidase).

## 2. Material and Methods

### 2.1. Oat Material Hybridization and Extraction

Oat material was obtained from the National Institute for Agricultural Research (INRA) in Rabat, Morocco. Accessions of tetraploid *A. murphyi dom* collected in different regions of Morocco, were involved in interspecific crosses with the Moroccan cultivar of *A. sativa* (Amlal). Amlal (Am) was used as female parent in the first crossing cycle. The yielded hybrids were backcrossed to their hexaploid parents respectively (*A. sativa* × *A. murphyi dom* × *A. sativa*). Ploidy analysis of the derivative hybrids was analyzed and only hexaploid hybrids (2*n* = 6× = 42) were selected and had been subjected to pedigree selection until reaching genetic stability. The Oat varieties (Amlal and F11-5) have been grown under the same conditions in Marchouch experimental station (68 km from Rabat), at 410 m of altitude, 498.3 mm of average annual rainfall, and a black crumbling soil. Oat varieties have been harvested in May 2013. The grain of each variety was cleaned and stored for evaluation. Oats phenolic extraction and quantification has been performed as previously described [10].

### 2.2. Chromatographic Analysis

α-Tocopherol analysis was performed using HPLC-FD (High Performance Liquid Chromatography-Fluorescence detector) equipped with a Zorbax SB-C18 column (Agilent Technologies, Palo Alto, CA, USA), using a fluorescence detector (excitation wavelength 290 nm, detection wavelength 330 nm) (Perkin Elmer, Monza, Italy).

The LC–DAD/ESI-MS (liquid chromatography-diode array detection/electrospray ionization mass spectrometry) system consisted of a binary pump (G1312A, Agilent Technologies, Inc., Wilmington, DE, USA) and an autosampler (G1330B, Agilent Technologies, Inc., Wilmington, DE, USA) coupled to a mass spectrometer equipped with an electrospray ionizer source (MS; ESI-; Micromass Quattro Micro; Waters, Milford, MA, USA). Reversed phase HPLC separation was carried out using a zorbax C18 column Zorbax (100 mm × 2.1 mm × 1.7 µm, Agilent Technologies, Santa Clara, CA, USA). The mass spectrometer was operated in negative ion mode with the following parameters: capillary voltage, 3.0 kV; cone voltage, 20 V; and extractor, 2 V. Source temperature was 100 °C, desolvation temperature was 350 °C, cone gas flow was 30 L/h, and desolvation gas flow was 350 L/h. The mobile phase components were 0.1% formic acid (A) and acetonitrile with 0.1% formic acid (B). The mobile phase gradient was: 0 min, 90% A; 0–18 min, 30% A; 18–20 min, 30% A; 20–23 min, 30% A; 23–25 min, 90% A; 25–30 min, 90% A. The injection volume was 10 µL and the column temperature was 35 °C. The flow rate of the mobile phase was 0.5 mL/min. The phenolic acids were identified on the basis of their retention times, MS spectra and molecular-ion identification.

### 2.3. Antioxidant Effect in Tetrahymena pyriformis Cell Culture

*T. pyriformis* is a suitable experimental organism for wide spectrum of functional, pharmacological studies allowing the use of indices common in animal studies, such as growth arrest and metabolic inhibition, kinetics and synthesis of specific molecules or enzymes. In this assay *T. pyriformis* was used as a cellular model to follow the protective effect of natural products against Hydrogen peroxide (H_2_O_2_)-induced OS. The ciliated protozoa *T. pyriformis* was grown axenically, without shaking, in the Proteose-peptone yeast Glucose defined medium (PPYG), as described by Mori [21]. The cells were incubated at 28 °C in capped 500 mL Fernbach flasks containing 100 mL of PPYG medium. For the bioassays, the *T. pyriformis* cultures were always in exponential growth phase, and they were adjusted to a density of 10^4^ cells/mL in fresh PPYG medium just before treatment with the tested chemicals. Protective antioxidant effect has been evaluated on *T. pyriformis* under H_2_O_2_ treatments in the presence of Oats extracts (controls were performed without H_2_O_2_). For this purpose, the 50% inhibitory concentration values (IC_50_) of tested substances (H_2_O_2_, Am and F11) were determined previously on *T. pyriformis* by cell counting. Treatments solutions were prepared in deionized water, just before testing; pH was checked, and readjusted to 6.5 if necessary. The IC_10_ of Oat extracts has been used for protective effects of Oat extracts simultaneously with H_2_O_2_ cytotoxicity under 75 µM doses. To evaluate cell viability, aliquots of 1 mL were taken from untreated and treated *T. pyriformis.* The samples were diluted in distilled water, and fixed with neutral buffered formalin containing 10% (*v*/*v*) formalin in Isoton buffer for 1 h. The cell number was determined with a hemocytometer under an optical microscope. To determine cell viability, a counting without fixation was realized in order to identify immobile and mobile cells (dead and live cells, respectively). The MTT assay [22] using the MTT reagent (3-(4,5-dimethylthiazol-2-yl)-2,5-diphenyltetrazolium bromide) was used to evaluate the effects of treatments on cell proliferation and/or mitochondrial activity. Four hours later the MTT salts were reduced to formazan blue in the metabolic active cells by mitochondrial enzyme succinate dehydrogenase to form NADH (Nicotinamide Adenine Dinucleotide Hydrogen) and NADPH (Nicotinamide Adenine Dinucleotide Phosphate). Absorbance was read at 570 nm. Oat antioxidant protective effect has been evaluated by measuring the protein content and two antioxidant enzymes: total superoxide dismutase (T-SOD) and Catalase (CAT) in the *T. pyriformis* homogenate [23,24,25].

### 2.4. Digestive Enzymes Inhibition and Kinetics

The α-amylase inhibition assay was conducted according to Kee [26] work, with slight modifications. Briefly, 250 µL of sample was mixed with 250 µL of α-amylase (240 U/mL, in 0.02 M phosphate buffer solution (PBS), pH 6.9, with 0.006 M NaCl). After incubating at 37 °C for 10 min, 250 µL of 1% (*w*/*v*) soluble starch (in 0.02 M PBS, pH 6.9) was added and the mixture was further incubated at 37 °C for 30 min, followed by adding 250 µL of dinitrosalicylic acid color reagent (DNS 96 mM, 30% Na-K tartrate, 0.4 M NaOH), and stopped by heating in a boiling water bath for 10 min. After cooling to room temperature, the mixture has been diluted with 2 mL of PBS and the absorbance was measured at 540 nm.

The α-glucosidase enzyme (0.1 U/mL) and substrate *p*-Nitrophenyl-α-d-glucopyranoside (*p*-NPG, 1 mM) were dissolved in PBS (0.1 M, pH 6.7), and all samples were dissolved in distilled water. The inhibitor (150 μL) was pre-incubated with the enzyme (100 μL) at 37 °C for 10 min, and then the substrate (200 μL) was added to the reaction mixture. The enzymatic reaction was performed at 37 °C for 30 min. The reaction was then terminated by the addition of Na_2_CO_3_ (1 M, 1 mL). All samples were analyzed in triplicate with different concentrations to determine the IC_50_ values, and the absorbance was recorded at 405 nm. The inhibition percentage (%) was calculated by the following equation for both assays:

$$\mathrm{Inhibition}\;\left(\%\right)\;=\frac{\left(\mathrm{AC}-{\mathrm{AC}}_{b}\right)-\left(\mathrm{AS}-{\mathrm{AS}}_{b}\right)}{\mathrm{AC}-{\mathrm{AC}}_{b}}\times 100$$

Absorbances are abbreviated as follows: AC (control), AC_b_ (control blank), AS (sample) and AS (sample blank).

The mode of inhibition of α-amylase and α-glucosidase was investigated with increasing concentrations of the substrate (Starch and *p*-NPG) in the presence of different concentrations of extracts. Then, the type of inhibition was determined by Lineweaver–Burk plot analysis of the data, calculated from the results according to Michaelis–Menten kinetics.

### 2.5. Anti-Hyperglycemic and Antioxidant Effect in Diabetic Model

#### 2.5.1. Animals

Wistar rats weighing (250–300 g) and Swiss albino mice (20–25 g), are bred in the central animal facility of the Faculty of Medicine and Pharmacy of Rabat, Morocco. Animals were kept in cages under standard laboratory conditions with tap water and standard diet *ad libitum*, in a 12 h light/12 h dark cycle at a temperature of 21 to 23 °C.

Ethics approval was also obtained from Mohammed V University in Rabat, under the responsibility of the Central Animal Facility and the Laboratory of Pharmacology and Toxicology at the Faculty of Medicine and Pharmacy of Rabat (01DEC2015). The experiments were conducted in accordance with the accepted principles outlined in the “Guide for the Care and Use of Laboratory Animals” prepared by the National Academy of Sciences and published by the National Institutes of Health and all efforts were made to minimize animal suffering and the number of animals used.

#### 2.5.2. Acute Toxicity

Acute oral toxicity study was performed as per 425 guidelines (OECD) from the Organization of Economic Co-operation and Development. Four groups of male Swiss albino mice (*n* = 6), each one selected by random sampling technique, were used for acute toxicity study. The animals were kept fasting overnight providing them only with water. Subsequent to the administration of extracts (2000 mg/kg), the animals were observed closely for the first 3 h in order to detect any toxic manifestations such as increased locomotors activity, salivation, clonic convulsion, coma and death. Subsequent observations were made at regular intervals for 24 h. The animals were observed for a further two weeks.

#### 2.5.3. Experimental Diabetes

Overnight fasted rats (OFR) were treated with nicotinamide (110 mg/kg, i.p.). Streptozotocin (65 mg/kg, i.p.) was injected 15 min after nicotinamide administration, in all groups except for normal control [27]. Animals were fed with glucose solution (5%) for 12 h to avoid hypoglycemia. Hyperglycemia was confirmed three days later, and the steady state of hyperglycemia was reached after 10 days. Serum glucose was determined by the glucose oxidase peroxidase method using a glucometer (One Touch Ultra, LifeScan, Milpitas, CA, USA). Animals having serum glucose between 200–300 mg/dL were selected for the study.

#### 2.5.4. Experimental Design

After establishment of the diabetic rats model, animals were divided into five treatment groups: Normal control (NC; 1 mL DW/200 g), Diabetic control (DC; 1 mL DW/200 g), Oat treatment (Am and F11; 2000 mg/kg) and Metformin (Met; 300 mg/kg). The dose was selected based on the results of our preliminary oral glucose tolerance tests (OGTTs) assays, in which we found that the effect of the 2000 mg/kg treatment was significantly higher than 500 mg/kg (*p* < 0.05) (data not shown). The animals received the respective treatment for 42 days (6 weeks). Blood was collected at the end of the experiment for hematological and biochemical analysis. Liver and kidney homogenate were analyzed for oxidative stress markers. Pancreas has been used for the histological characterization. OGTTs were performed on day 1 and 40 from the beginning of the experiment. During the study period of 42 days, the rats were weighed daily using electronic balance, and glycaemia levels were recorded weekly. Food and water intake, and urinary volume were determined for the first and last day before OGTT using metabolic cages.

#### 2.5.5. Oral Glucose Tolerance Test

OFR were administered with glucose (2000 mg/kg) orally by means of gastric intubation. Animals in Am and F11 groups were administered orally with Oat extracts, at a dose of 2000 mg/kg, 30 min before the oral administration of glucose. Whereas animals in group Met were given metformin (300 mg/kg). The animals of control i.e. group NC and DC were given orally equal volume of water only. Blood samples were collected from the tail vein at 0, 30, 60, 90, 120 and 150 min. Total glycemic responses to OGTT were calculated from respective areas under curves (AUC) of glycaemia during the 150 min observation period. The Δ variation of glycaemia was defined as the difference between glycaemia at t_0_ and a following time point.

#### 2.5.6. Behavioral Assays

The elevated plus maze (EPM) is an ethological model of anxiety in rodents. After 34 days of treatments, OFR have received their treatments at the fixed doses. Thirty minutes after administration, each rat was placed in the central square facing an open arm and allowed to freely explore the maze for 5 min. The following measurements were recorded; arm entries, total time spent in open arms and total number of entries in open arms [28]. Two days later (day 36), the OFR have received their treatments at the fixed doses, and thirty minutes later, the animals’ spontaneous activity was evaluated in an open field test (OFT). In individual tests, the rats were placed at the same point and allowed to freely explore the apparatus for 10 min. The following measurements were then recorded; total squares entries, central squares entries and time spent in central squares [29].

#### 2.5.7. Hematological and Biochemical Analysis

On day 42, blood samples were collected from the Jugular vein by using capillary tubes containing Ethylenediaminetetraacetic acid (EDTA) (anti-coagulant). The following hematological parameters were evaluated in the collected blood samples: Total hemoglobin (HGB), red (RBC) and white (WBC) blood corpuscles count, neutrophils, lymphocytes, eosinophils, monocytes, basophils and platelet count using fully automated analyzer (Architect c8000, Clinical Chemistry System, Chicago, IL, USA). Biochemical parameters *viz*. aspartate aminotransferase (AST), alanine aminotransferase (ALT), Total proteins, urea, uric acid, creatinine, cholesterol, triacylglycerols (TG), high density lipoprotein (HDL), low density lipoprotein (LDL), lactate dehydrogenase (LDH), Sodium, Potassium and Chlorine were determined using the same analyzer. The atherosclerosis index (AI) was calculated as LDL/HDL ratio.

#### 2.5.8. Key Enzymes and Markers of Oxidative Stress

After sacrifice, liver and kidney were dissected out, and a 10% of organs homogenate was prepared in ice-cold PBS 50 mM using Teflon glass homogenizer. The homogenate was centrifuged at 3500 rpm for 10 min (at 4 °C) using cooling centrifuge (Mikro 220R, Hettick Lab Technology, Tuttlingen, Germany). The pellet was discarded and supernatant obtained was used for the estimation of antioxidant enzymes, oxidative stress markers and protein content in the homogenate.

The protein content was estimated following the method of Lowry [23]. The antioxidant enzymes were expressed as μmol/min/mg protein. Catalase (CAT) activity was determined according to the method of Aebi [30]. T-SOD activity was determined by the method of Beauchamp and Fridovich [22]. The MnSOD activity was measured after addition of 2 mM KCN in the solution. The CuZn-SOD activity was calculated by subtraction as follows:

CuZn − SOD = T-SOD − Mn-SOD

Activities of Glutathione peroxidase (GPx) were estimated according to the method of Mannervik [25]. Glutathione (GSH) was estimated by a previously described method [31], and expressed as μmol GSH/mg protein using GSH standard. Thiobarbituric acid reactive substances were estimated as malondialdehyde (MDA) equivalent (nmol MDA/mg protein), from the calibration curve [32].

#### 2.5.9. Histological Study of Pancreas

The pancreas from each rat was fixed in 10% buffered formalin and processed via classical histology method using the paraffin wax embedding techniques (dehydration, clearing and embedding). The paraffin embedded-sections were cut at 4 μm thickness using microtome (Shandon Hypercut, Runcorn, UK), and then stained by hematoxylin and eosin staining method. Stained sections of pancreas were qualitatively (morphological) analyzed on microscope (Leica Microsystems DM2500, Wetzlar, Germany), and photomicrographs of histological alterations have been taken at ×400 magnification.

### 2.6. Statistical Analysis

Data were expressed as the mean values ± standard deviation (SD) for each measurement. The data were also analyzed by one-way analysis of variance (one-way ANOVA). Post Hoc procedure was used for significance of difference (*p* < 0.05). Analysis was performed with Graph pad prism 6.0.

## 3. Results

### 3.1. Chemical Analysis

Chemical analysis of Oat varieties (Table 1) revealed the phenolic composition of Oat extracts. The *p*-Hydroxybenzoic acid constitute the major phenolic compound in both extracts with 1840.34 ± 30.45 mg/Kg for Amlal and 1270.02 ± 38.34 mg/Kg for F11, followed by the Syringic acid (1830.66 ± 90.21 mg/Kg for Amlal and 310.41 ± 33.09 mg/Kg for F11), and the Caffeic acid (250.67 ± 32.11 mg/Kg for Amlal and 421.54 ± 12.32 mg/Kg for F11). Significant contents of Sinapic, Ferulic, Gallic, *p*-Coumaric and Chlorogenic acids were found. α-Tocopherol contents in Amlal and F11 were 1.65 ± 0.22 and 1.82 ± 0.12 mg/kg in Amlal and F11 respectively.

### 3.2. Antioxidant Effect in T. pyriformis

**Figure 1:**
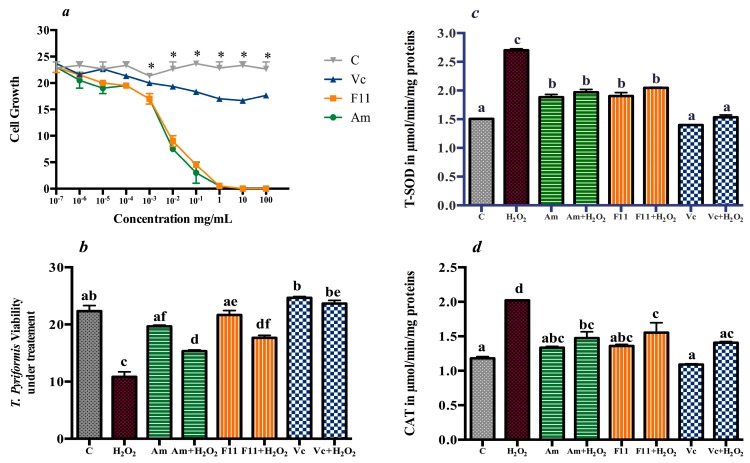
Oat protective effect on antioxidant enzymes under oxidative stress in *T. pyriformis.* (**a**) Oat and Vitamin C effect on *T. pyriformis* growth. (**b**) *T. pyriformis* viability under H_2_O_2_ treatment in the presence of extracts. (**c**) T-SOD levels *T. pyriformis* cultures. (**d**) CAT levels *T. pyriformis* cultures, Data are reported to mean (*n* = 3) ± SD. Values not sharing a common letter (a–d) differs significantly at *p* < 0.05. (*) differ significantly from the control.

Different concentrations of Oat extracts and H_2_O_2_ were screened to determine their cytotoxicity (IC_50_) on *T. pyriformis*. The determined IC_50_ of H_2_O_2_ (0.75 mM) was used to establish the oxidative stress environment and the IC_10_ of Oat varieties (1 μg/mL) were used for antioxidant treatment in comparison with vitamin C at the same dose (Figure 1).

H_2_O_2_-induced stress reduced *T. pyriformis* viability by 48.49% compared to the control group. Cell viability in treated groups was improved by 20.15% in Am, 30.60% in F11 and 57.47% in Vc compared to H_2_O_2_ group. Antioxidant enzymes, including T-SOD and CAT levels increased significantly by 38.92% and 36.04% in H_2_O_2_ compared to the non-treated control (*p* < 0.05). However Oat treatment reduced the level of the expression of those enzymes. T-SOD release was reduced by −20.16% in Am, −17.80% in F11, and −37.77% in the positive control (Vc). Similarly, the treatments decreased CAT levels by −20.14% in Am, −15.80% in F11 and −23.77% in Vc.

### 3.3. In Vitro Inhibition of Digestive Enzymes

In an array to explore the in vitro antidiabetic activity, Moroccan Oat extracts were screened for the α-amylase and α-glucosidase inhibitory properties. Effects were compared with the commercially available α-glucosidase inhibitor, acarbose (Ac).

**Figure 2:**
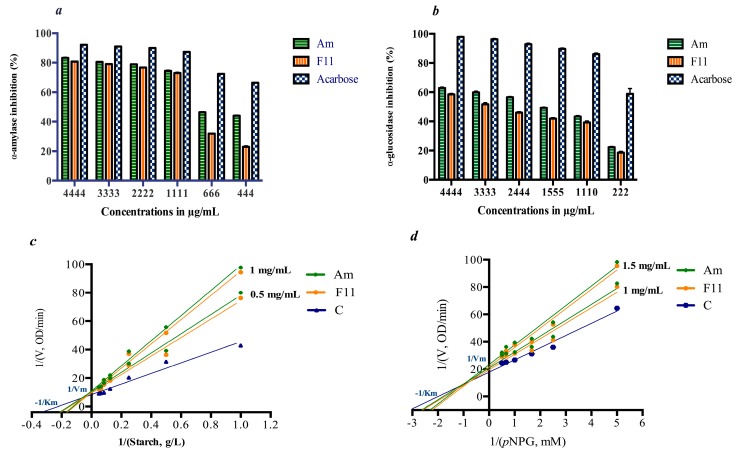
α-amylase and α-glucosidase inhibitory activities. (**a**) α-amylase inhibitory activities of Oat extracts. (**b**) α-glucosidase inhibitory activities of Oat extracts. (**c**) Lineweaver burk plot of Oat α-amylase inhibition. (**d**) Lineweaver burk plot of Oat α-glucosidase inhibition, Data are reported to mean (*n* = 3) ± SD.

Inhibitory activities of the extracts were evaluated at different concentrations and results were given in Figure 2. Acarbose and extracts showed a dose dependent inhibitory effect on enzymes. The IC_50_ values for anti-amylase and anti-glucosidase, activity of Am and F11 were lower than that of acarbose, a very well known drug with well-established activity (Figure 2). IC_50_ for amylase inhibition were 723.91 μg/mL and 1027.14 μg/mL for Am and F11 respectively. While, acarbose inhibitory concentration was 396.42 μg/mL. α-glucosidase inhibitory effect of Oat extracts was much more lower than its equivalent of acarbose. In term of IC_50_, significant differences have been registered between Oat varieties and acarbose: Am (1548.12 μg/mL), F11 (1803.52 μg/mL) and Ac (199.53 μg/mL).

A Lineweaver-Burk double reciprocal plot of enzymes activity in the presence of Oat extracts as inhibitor was plotted. Km and Vmax values increased with increasing Oat concentrations. The kinetic study (Figure 2) suggests that Oat extracts metabolites intersected in the second quadrant indicating a mixed-type of inhibition in both enzymes (α-amylase and α-glucosidase).

### 3.4. Anti-Hyperglycemic and Antioxidant Effect in Diabetic Model

#### 3.4.1. Acute Oral Toxicity and Metabolic Parameters

Animals treated with Oat extracts did not show any change in their behavioral pattern during the acute toxicity study. There was no significant difference in the body weight and food consumption when compared to the vehicle treated group. Also, no apparent pathological changes were seen. Thus, it was concluded that Oat extracts were safe at 2000 mg/kg.

Table 2 shows the effect of daily administration of Oat extracts on body weight, urinary volume, and food and water intakes in diabetic rats. After six weeks of treatment, DC rats showed a considerably reduction of body weight by 50.91% compared to the initial value. The administration of Oat extracts (Am and F11) resulted in significant weight gain as compared to DC (40.47%, 30.07% and 39.01% for Met, Am and F11 respectively). In the other hand, food intake increased by 29.47% in DC, 11.99% in Met, 19.29% in Am and 26.58% in F11. Also, the elevated water intake and urinary volume (139.43–56.11%) in DC was significantly reduced to (−18.95%, −18.68%) in Met, (−45.96%, −39.33%) in Am and to (−62.57%, −39.20%) in F11. Compared to DC, urinary volume, food and water intake in treated groups has decreased, while body weight has increased significantly (*p* < 0.05).

#### 3.4.2. Glucose Tolerance and Anti-Hyperglycemic Effect

**Figure 3:**
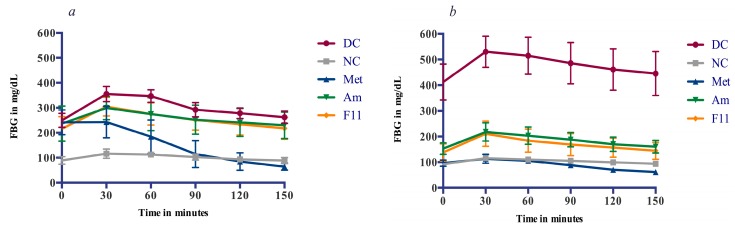

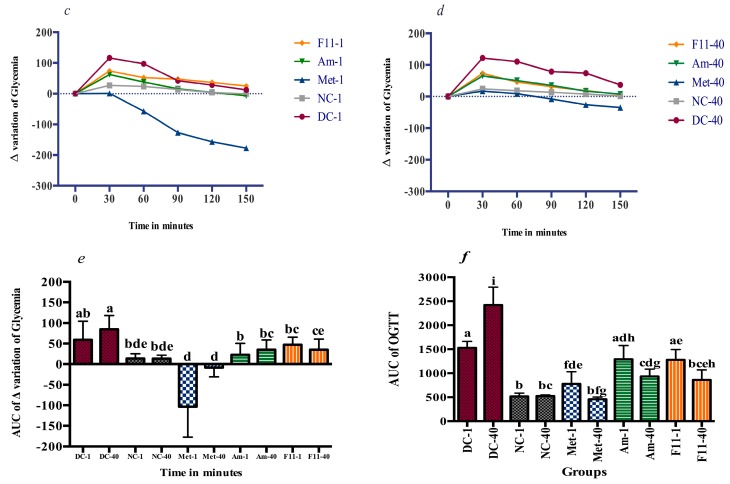
Oral glucose tolerance before and after treatment period. (**a**) OGTT of the first day 1; (**b**) OGTT of the day 40; (**c**) Δ variation of glycaemia during OGTT-Day 1; (**d**) Δ variation of glycaemia during OGTT-Day 40; (**e**) AUC of Δ variation of glycaemia during OGTT challenge; (**f**) AUC of OGTT, Data are reported to mean (*n* = 8) ± SD. Values not sharing a common letter (a–i) differs significantly at *p* < 0.05.

In order to evaluate the effectiveness of each treatment, OGTT was performed at the beginning and the end of the study. OGTT revealed that blood glucose reached its peak level at 30 min after the glucose load in all groups before and after treatments (Figure 3). The initial administration of Oat extracts (Am and F11) at 2000 mg/kg did not significantly reduce the AUC of blood glucose levels compared to the vehicle in DC. However, as expected Metformin show decrease (−52.48%) in glucose levels and stabilize glycaemia to normal values in diabetic rats, 90 min after administration.

Across the six weeks of treatment period, FBG has been shown to be decreased gradually in Am, F11, and Met groups (Table 2). Glycemic levels in Oat-treated groups decreased significantly (−35.57% and −36.25%) compared to the dramatically increase (64.97%) in DC. Metformin has been shown to possess largely stronger effect (−60.28%), stabilizing FBG to its normal levels (Table 1).

At the end of treatment, the beneficial effects of Oat extracts on glucose metabolism of diabetic rats become evident. In the OGTT, after 30 min of the glucose overload, treated groups (Am, F11 and Met) were able to reduce glycaemia (Δ variation of glycaemia) more efficiently (65.33, 73.14 and 17.14 mg/dL) than DC (118 mg/dL). To the end of the challenge test, Oat extracts reduced blood glucose level significantly compared to DC. Hence, the glucose tolerance was significantly improved after Oat supplementation and metformin treatment.

#### 3.4.3. Hematological and Biochemical Indices

In order to evaluate the protective effect of the above treatments on physiological functions, the hematological and biochemical parameters were analyzed (Table 3). The total hemoglobin, red blood corpuscles count, neutrophils, lymphocytes, monocytes and eosinophils were not significantly different between the evaluated groups (Table 3). However, the white blood corpuscles count increased significantly in DC (*p* < 0.05). Moreover, the ALT and AST in Am, F11 and Met treated rats decreased significantly (*p* < 0.05) as compared to diabetic animals (Table 3). Nevertheless, total serum protein did not show any significant change in DC compared to NC. In the other hand LDH and urea in Oat and Met has shown decreased levels as compared to DC (*p* < 0.01). Serum creatinine did not show significant variations among groups. In the lipid profile analysis, the diabetic animals showed significant increase (*p* < 0.01) in the total cholesterol and TG; simultaneously a decrease in HDL compared to normal and treated rats. LDL levels did not show significant variations between DC and NC. Atherosclerosis index demonstrated significant differences in all groups (*p* < 0.05).

#### 3.4.4. Oxidative Stress Markers and Antioxidants Enzymes

By the end of the treatment period, oxidative stress markers (MDA and GSH) and antioxidant enzymes (T-SOD, Mn-SOD, CuZn-SOD, CAT and GPx) were analyzed and results are presented in Table 4. Lipid peroxidation products as expressed in MDA equivalents in diabetic non-treated animals were much higher than those found in NC and treated rats. MDA levels at the liver was significantly higher compared to NC (*p* < 0.001), while treatment registered a lesser increase in Met (30.95%), Am (154.76%) and F11 (407.14%). Likewise, at the kidney level, similar increase in MDA has been registered (405.49%). Treatments of diabetic groups decreased the MDA production in Met (−274.72%), Am (−378.01%) and F11 (−256.58%) compared to DC. The T-SOD, Mn-SOD and CuZn-SOD levels in DC have increased significantly compared to NC and treated groups (*p* < 0.05). T-SOD increased by 124.51% in DC compared to NC at the liver, while in the kidney the increase was less marked by 52.16%. Am and Met treatments decreased T-SOD and similarly Mn-SOD and CuZn-SOD in both organs (*p* < 0.05). Also, compared to NC, diabetic animals have shown a significant increase (*p* < 0.05) of CAT by 218.68% in the liver and 50.05% in the kidney (Table 4). The treated groups exhibited a significant decrease (*p* < 0.05) in CAT levels except for the F11 group. Oat extract and metformin induced a reduction of CAT peaks in treated diabetic animals (−194.63%, −170.41% and −63.66% for Met, Am and F11 respectively) in the liver and similarly in the kidney. Moreover, DC rats were also characterized by an increase in GSH by 85.59% in the liver. Administration of the treatments induced a significant (*p* < 0.05) decrease of GSH in the liver for Met (−37.28%), Am (−55.08%) and F11 (−42.36%), compared to DC. The kidney levels of GSH followed the same tendency. GPx Levels in the other hand increased significantly in liver of the diabetic controls (123.60%). Treated groups (Am and Met) show decreased values compared to diabetic control at the liver level, except for F11 group who did not show significant variations in comparison with DC. GPx levels in the kidney did not show significant differences.

#### 3.4.5. Behavioral Analysis

As depicted in Table 5 the behavioral analysis of DC based on the EPM demonstrated a significant decrease in arm entries (−90.99%), open arms entries (−89.18%), and in time spent in open arms (−87.29%) compared to NC. Similarly, the OFT parameters of DC show significant decrease in total squares entries (−71.14%), central squares entries (−89.10%) and time spent in central squares (−62.21%), compared to NC. Metformin and Oat treatments resulted in significant increase of locomotor and exploratory activities in both assays (*p* < 0.05).

#### 3.4.6. Pancreas Histopathology

**Figure 4:**
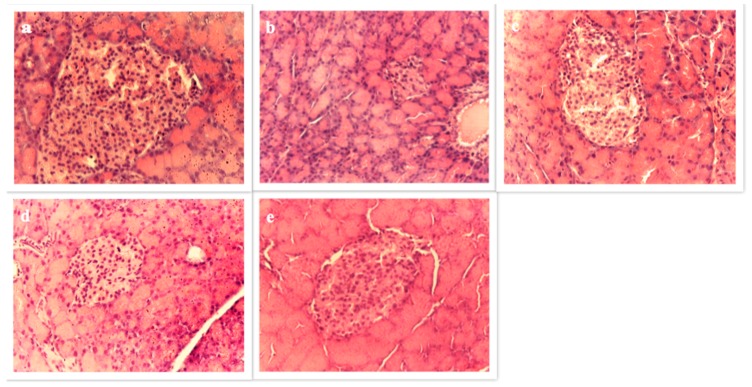
Effect of diabetes and oat treatment on histological alterations in the pancreas. Representative photomicrographs of histological alterations stained with hematoxylin and eosin at ×400 magnification. Normal control (**a**); Diabetic control (**b**); Metformin (**c**); Amlal (**d**); F11-5 (**e**).

The pathologic features of pancreas tissues in each group are shown in Figure 4. Normal control showed normal pancreatic parenchyma cells and islet cells (round or oval-shaped cell mass) whereas DC showed focal necrosis and dilated acini. Metformin and Oat-treated groups showed minimal pathological changes.

## 4. Discussion

### 4.1. In Vitro Antidiabetic Activity

This study reports the inhibitory kinetics of Oat aqueous extracts against key enzymes linked to hyperglycemia. The in vitro assay demonstrated a moderate inhibitory effect compared to the positive control. Regarding the safe profile of Oat extract, it could be of interest to consider Oat formulation for inhibition of digestive enzymes in the small intestine. Oat mixture can display an antidiabetic effect if used as a capsulated complement. The presence of numerous phenolic compounds among the extract mixture e.g., *p*-Hydroxybenzoic, Syringic and Caffeic acids, resulted in a mixed type of inhibition. Previously, the association of several bioactive compounds revealed to be much more effective and safe in therapeutic strategies [33].

In fact, the heterogeneous character of type 2 diabetes results from the dynamic interaction between defects in insulin secretion and insulin action. Such a deficiency results in increased concentrations of blood glucose, which in turn damages many of the body’s systems [34]. Therefore, there is a need to control postprandial blood glucose for diabetes management. One of the strategies in glucose control is the inhibition of digestive enzymes [17], such as α-glucosidase and α-amylase, which results in a significant reduction of the post-prandial blood glucose conferring an important target for diabetes management [35]. Although the wide availability of glucosidase inhibitors, many works sought natural sources in the hope to present nutritional alternatives with minimal side effects and low therapy costs [36]. Actually, a pseudo-tetrasaccharide of microbial origin namely acarbose, inhibits the brush-border enzymes glucoamylase, dextrinase, maltase and sucrase as well as the pancreatic α-amylase [17]. Additionally, other digestive enzymes inhibitors [37] are available (miglitol, emiglitate voglibose). Despite the effectiveness of acarbose as antidiabetic drug with glucosidase inhibitory properties, food alternatives are indeed needed because of acarbose side effects such as diarrhea and flatulence that occurs when colonic bacteria ferment the undigested carbohydrates, resulting in gas formation [38]. Also uncommon hepatotoxicity cases have been registered following long-term intake [39]. Currently, there is renewed interest in nutritional therapies and functional foods with preventive effects on diabetes and obesity [40]. Few papers described the Oat glucosidase and amylase inhibitory activities [16,17]. Nevertheless, none of the previous studies has determined the inhibitory concentrations and the kinetic of inhibition. Also the reported works focused on whole oatmeal and did not especially study the aqueous phenolic rich extract. Oat anti-enzymatic activity is probably linked in part to its phenolic content. As we have adopted a complementary nutritional therapy with multi-target bioactivities (antioxidant and antidiabetic), the safe Oat phenolic extracts have been chosen for in vivo and in vitro activities. Inhibitory effect of hybrid and parent Oat varieties was lower than acarbose, especially for glucosidase inhibition. As a complex mixture, Oat extracts exhibited a mixed inhibition, which is an intermediate mode between the competitive and uncompetitive inhibition [41]. The extracts metabolites were able to bind either to the free enzyme or the enzyme-substrate complex. Phenolic compounds were reported to exhibit a glucosidase inhibitory effect. Especially cereal grains, such as Barley [42], Wheat [43] and Quinoa [44], were reported to inhibit intestinal glucosidase.

### 4.2. Oxidative Damage Prevention and Anti-Hyperglycemic Effect

Recently, we have reported the nutritional characteristics and biochemical composition of Moroccan Oat varieties [10]. This assay is an attempt to investigate the in vivo antidiabetic and antioxidant properties of the Moroccan Oat variety (Amlal) and its derivative new line (F11-5).

Oat plays a role in modulating the metabolic effects observed after fiber-rich meals. As a soluble fiber with viscous characteristics, Oat β-glucans modifies properties of chyme in the upper part of the gastrointestinal tract affecting gastric emptying, gut motility, and nutrient absorption, which are reflected in lower postprandial glycemic and insulin responses [45]. Thus, oat β-glucan intake is beneficial for healthy subjects and patients with type-2 diabetes [46]. Manifestly, many papers described the Oat antidiabetic action linked to its fiber content and viscosity [47]. Moreover, Oat antioxidant effect is well described in literature using in vitro methods [10]. However few papers discussed the preventive effect of Oat extracts on hyperglycemia-induced OS. In our multi-target Oat-therapy approach, we tried to focus on both diabetes and oxidative stress. In this regard we have decided to use the aqueous phenolic extracts instead of other Oat extracts. The in vivo antioxidant and antidiabetic activities were evaluated in male wistar rats, following acute toxicity study in female Swiss mice. The use of female in toxicity studies is mainly due to their higher sensitivity. According to the OECD 425, the preferred rodent species is the rat, although other rodent species may be used. On the other hand, for diabetes models, several animal species, including the mouse and rat are sensitive to the pancreatic β-cell cytotoxic effects of Streptozotocin (STZ). However, some mice strains are less sensitive to this toxin.

In summary, the results from in vivo study of Oat extract intake, revealed an amelioration of glucose tolerance, a decrease in FBG and an antioxidant effect based on oxidative stress markers expression compared to diabetic stressed control. Furthermore, Oat intake prevented anxiety like behavior, hypercholesterolemia and atherosclerosis in diabetic rats.

Animal models of diabetes are an important tool for in vivo screening of antidiabetic compounds. The fungal compound streptozotocin is a hydrophilic nitrosourea analogue with antibiotic and chemotherapeutic activities. Owing to its glucose like similar structure, it enters β-cells via GLUT2 (Glucose transporter 2) transporters in a similar way to glucose [48], resulting in DNA alkylation, nitric oxide release and ROS generation leading to insulin reduced synthesis and diabetogenic state. In the STZ-NA (Streptozotocin-Nicotinamide) model of diabetes the use of NA a poly-ADP-ribose (Poly-Adenosine diphosphate-ribose) synthetase inhibitor, is mainly for its protective effect on β-cells function via the prevention of reduction in the level of nicotinamide adenine dinucleotide; thereby it results in a reverses of the insulin secretion inhibition lowering the degenerescence in the experimental model following the STZ administration, which show similar characteristics to T2DM (Type 2 Diabetes Mellitus) [49].

Actually, we have demonstrated that Oat extracts at 2000 mg/kg reduced significantly the FBG in STZ-NA-induced diabetic rats after 6 weeks of treatment. The OGTT performed at the beginning and the end of the treatment period show significant improvement in impaired glucose tolerance. Body weight loss in diabetic animals has been attributed to the waste in muscle mass [50]. Oat treatment and anti-hyperglycemic effect resulted in a decrease of body weight loss frequency. In the other hand, the increase in urinary volume and water and food intake in diabetic animals is comparable to polyphagia and polydipsia observed in diabetic patients [51]. Oat treatment prevented those manifestations and decreased diabetic symptoms impact. The decreased serum glucose levels after treatment period can probably be linked to Oat ability to inhibit digestive enzymes at the intestinal level. Based upon histopathological results it can be also hypothesized that Oat may probably exhibit protective effect on pancreatic β-cells against STZ toxic effects. Manifestly, β-cells number and size in treated animals support this hypothesis. Furthermore, diabetes complications such as hypercholesteremia and hypertriacylglycerolemia are primary factors involved in the development of atherosclerosis and coronary heart disease [52]. Oat treatment significantly reduced serum TG and total cholesterol in STZ diabetic rats. Consequently, it is suggested that Oat intake and Met treatment could modulate blood lipid abnormalities and reduce atherosclerosis risk. As a vital organ the liver metabolizes nutrients and detoxifies harmful substances. Accordingly, ALT and AST are reliable markers of liver function [53]. In the STZ-NA model of diabetes the increase in ALT and AST activities in plasma indicates the STZ hepatotoxic effect and the liver necrosis [54]. Am and F11 treatments reduced these enzymes levels in plasma compared to the DC and consequently prevented the liver damage; which indicates their hepatoprotective effect. The positive control group has been treated by metformin, which is a guanidine-containing compound currently recommended as first line therapy for all newly diagnosed T2DM patients [51]. Metformin decrease glucose production in the liver via the suppression of gluconeogenesis. Moreover, enhance the insulin suppression of endogenous glucose production and, to a lesser extent, reduce the intestinal glucose absorption and probably improve the glucose uptake and utilization by peripheral tissues [55]. Indeed, there is a growing evidence that hyperglycemia causes OS in a variety of tissues through ROS production [56]. At the same time, free radicals and ROS are implicated in the physiological gluco-regulatory systems. For instance, insulin release that is stimulated by glucose production in β-cells has been linked to H_2_O_2_ regulatory action [57]. Recent mathematical modeling described the OS dynamics to be of oscillatory nature [58] and tissue depending [59], which may explain the inconsistency of experimental data related to antioxidant enzymes measurements. Alternatively, focusing on stable OS markers such as MDA may reflect more reliable information on OS damage under long-term dynamics. Correspondingly, antioxidant enzymes and thiols form the first line of defense against ROS in the cells. In fact, SOD activity enhances the spontaneous dismutation of superoxide radicals to H_2_O_2,_ before it is removed by CAT activity. In contrast to CAT, the GPx act by reducing lipid hydroperoxides to the corresponding alcohols and reduce free H_2_O_2_ to H_2_O even at low concentrations. Moreover, the major endogenous antioxidant GSH directly neutralizes the free radicals and ROS, and maintains the exogenous antioxidants such as vitamins C and E in their reduced forms [60]. The increase in antioxidant enzymes activity in various tissues could reflect an adaptation to diabetes-induced OS. Our results showed that the level of MDA and GSH and the activities of T-SOD, Mn-SOD, CuZn-SOD, and CAT in the liver and kidney of diabetic rats significantly increased when compared with the normal control animals. However GPx increased only in the liver. The change in the antioxidant enzymes activities at the kidney and liver, show similarities to those seen in rats subjected to food deprivation-induced weight loss [61]. The reduced lipid peroxidation products and antioxidant enzymes after treatment likely indicates that Oat extracts might be a good source of metabolites with protective effect against diabetic-induced oxidative stress complications. Similarly, our results demonstrated clearly the protective effect of Oat extract against H_2_O_2_ oxidative stress in the cellular model of *T. pyriformis*. Both models indicate the in vivo antioxidant effect of Oat extracts, which can be attributed to its main phenolic compounds. Many phenolics with reported antioxidant effect has been isolated and identified in Oat such as ferulic acid and avenanthramides derivatives [62]. The radical scavenging effect of those Oat metabolites is highly linked to their bioavailability [63] and biotransformation [64].

Diabetes and hyperglycemia-induced oxidative stress can result in neurological complications such as dementia and depression [14]. Also the diabetogenic agent streptozotocin when injected at the intracerebro-ventricular level in rats can produce an Alzheimer disease (AD) model that triggers an insulin resistant brain state [65]. The plausible explanation suggests that low metabolism of brain glucose associated with oxidative stress and neuro-inflammation can result in mitochondrial dysfunction and apoptosis at the hippocampus, leading to accumulation of β-amyloid plaques and neurofibrillary tangles. This oxidative, neuro-inflammatory and degenerative environment induces progressively the memory impairment [66]. Moreover, in previous reports [67], STZ-treated rats displayed increased anxiety-like behavior in different paradigms, such as the open-field test, and the elevated plus maze. In fact, metformin administration significantly attenuated the anxiety-like behavior [68]. In our assays, the Oat and metformin treatment significantly improved cognitive status and prevented anxiety-like behavior in diabetic animals.

## 5. Conclusions

The present study suggests that Oat extracts are able to prevent oxidative stress in the diabetic liver and kidney. Also it has been demonstrated that Oat antidiabetic activity is mediated by digestive enzymes inhibition including (α-amylase and α-glucosidase). Oat extracts have been shown to protect antioxidant systems under diabetic stress and to prevent lipid peroxidation. Furthermore, Oat treatment preserved body weight and ameliorated liver functions (ALT and AST), lipid profile (cholesterol, TG) and reduced atherosclerosis index in diabetic animals. Finally, Oat and metformin intake significantly improved the anxiety-like behavior in diabetic animals.

## Figures and Tables

**Table 1:** Phenolic and tocopherol composition of hybrid Oat and parent in mg/Kg.

Compounds	Amlal	F11-5
Gallic acid	41.08 ± 2.32 ^b^	13.34 ± 2.29 ^a^
Chlorogenic acid	2.56 ± 0.45 ^a^	1.78 ± 0.12 ^a^
*p*-Hydroxybenzoic acid	1840.34 ± 30.45 ^b^	1270.02 ± 38.34 ^a^
Caffeic acid	250.67 ± 32.11 ^a^	421.54 ± 12.32 ^b^
Syringic acid	1830.66 ± 90.21 ^b^	310.41 ± 33.09 ^a^
*p*-Coumaric acid	26.67 ± 3.22 ^b^	16.43 ± 1.90 ^a^
Ferulic acid	70.45 ± 1.87 ^b^	1.98 ± 0.49 ^a^
Sinapic acid	17.10 ± 2.09 ^b^	11.62 ± 2.78 ^a^
Salicilyc acid	4.67 ± 0.07 ^b^	1.78 ± 0.16 ^a^
α-Tocopherol	1.65 ± 0.22 ^a^	1.82 ± 0.12 ^b^

Data are reported to mean (*n* = 2) ± SD. Values in the same raw not sharing a common letter (^a,b^) differs significantly at *p* < 0.05.

**Table 2:** Changes in fasting blood glucose and metabolic parameters.

Measure		NC	DC	Met	Am	F11
**FBG (mg/dL)**	**D1**	89.15 ± 16.48 ^a^	249.85 ± 28.05 ^a^	246.16 ± 52.60 ^a^	236.71 ± 70.16 ^a^	215.80 ± 48.83 ^a^
	**D14**	86.83 ± 9.64 ^a^	295.50 ± 28.98 ^b^	107.66 ± 7.14 ^b^	216.42 ± 25.26 ^a^	193.14 ± 34.78 ^a^
	**D28**	91.83 ± 10.04 ^a^	381.80 ± 38.10 ^c^	100.50 ± 3.50 ^b^	189.85 ± 33.30 ^a^	176.28 ± 34.51 ^a^
	**D42**	92.16 ±7.19 ^a^	412.20 ± 49.87 ^c^	92.83 ± 9.78 ^b^	152.50 ± 22.38 ^b^	137.57 ± 33.57 ^b^
**Food intake (g)**	**D1**	16.85 ± 1.57 ^a^	19.14 ± 3.02 ^a^	18.85 ± 2.60 ^a^	19.28 ± 2.13 ^a^	18.28 ± 2.87 ^a^
	**D41**	19.42 ± 3.10 ^a^	27.14 ± 2.19 ^b^	21.42 ± 1.98 ^a^	23.00 ± 1.41 ^a^	23.14 ± 2.91 ^a^
**Water intake (mL)**	**D1**	24.71 ± 4.46 ^a^	71 ± 11.71 ^a^	57.28 ± 7.69 ^a^	67.14 ± 3.71 ^a^	63.00 ± 7.16 ^a^
	**D41**	25.14 ± 5.61 ^a^	170 ± 14.54 ^b^	68.14 ± 10.17 ^a^	98.00 ± 7.50 ^b^	102.42 ± 15.05 ^b^
**Urinary volume (mL)**	**D1**	10.57 ± 1.98 ^a^	45.57 ± 6.87 ^a^	43.57 ± 2.69 ^a^	37.42 ± 5.42 ^a^	39.71 ± 5.46 ^a^
	**D41**	11.14 ± 1.77 ^a^	71.14 ± 2.60 ^b^	51.71 ± 5.61 ^a^	52.14 ± 2.41 ^b^	55.28 ± 5.58 ^b^
**Body Weight (g)**	**D1**	208.57 ± 25.33 ^a^	241.85 ± 10.52 ^a^	221.71 ± 56.29 ^a^	221.42 ± 26.45 ^a^	214.85 ± 57.01 ^a^
	**D41**	236.16 ± 25.89 ^a^	118.71 ± 21.62 ^b^	198.57 ± 13.50 ^a^	175.28 ± 14.39 ^a^	189.28 ± 58.01 ^a^

Data are reported to mean (*n* = 8) ± SD. Values in the same column and category not sharing a common letter (^a–c^) differs significantly at *p* < 0.05.

**Table 3:** Hematological and biochemical analysis of diabetic animals.

Parameters	Unit	NC	DC	Met	Am	F11
**Hematology**						
HGB	g/dL	11.25 ± 0.49 ^a^	14.32 ± 1.27 ^a^	12.93 ± 0.98 ^a^	11.1 ± 3.39 ^a^	12.02 ±2.04 ^a^
RBC	10^−6^/uL	6.87 ± 0.02 ^a^	8.36 ± 0.59 ^b^	7.84 ± 0.21 ^ab^	12.76 ± 0.50 ^c^	6.76 ± 1.05 ^a^
WBC	10^−3^/uL	4.23 ± 0.89 ^a^	21.31 ± 3.69 ^c^	10.43 ± 5.43 ^ab^	18.05 ± 1.18 ^b^	12.83 ± 3.90 ^ab^
Neutrophils	10^−3^/uL	15.50 ± 4.65 ^a^	21.63 ± 6.29 ^ab^	16.43 ± 3.47 ^b^	15.83 ± 3.44 ^b^	17.01 ± 0.10 ^b^
Lymphocytes	10^−3^/uL	65.25 ± 15.34 ^abc^	54.05 ± 1.62 ^c^	45.65 ± 9.89 ^b^	73.93 ± 4.20 ^b^	55.33 ± 4.90 ^c^
Monocytes	10^−3^/uL	4.95 ± 0.91 ^a^	9.86 ± 1.77 ^d^	5.24 ± 1.69 ^c^	6.71 ± 1.65 ^b^	5.73 ± 0.49 ^bcd^
Eosinophils	10^−3^/uL	2.80 ± 0.97 ^ab^	2.77 ± 0.73 ^a^	1.98 ± 0.59 ^c^	3.91 ± 1.60 ^abc^	2.10 ± 0.81 ^bc^
Basophils	10^−3^/uL	n.d	n.d	n.d	n.d	n.d
Platelet count	10^−3^/uL	508 ± 89.78 ^ab^	516.75 ± 93.11 ^ab^	534.33 ± 51.18 ^a^	548.86 ± 41.71 ^ab^	584.50 ± 61.51 ^b^
**Liver function**						
ALT	IU/L	66.66 ± 12.01 ^ab^	130.05 ± 4.56 ^c^	84.66 ± 17.62 ^b^	68.28 ± 37.66 ^ab^	72.40 ± 18.14 ^a^
AST	IU/L	104.5 ± 14.86 ^ac^	225.66 ± 53.65 ^d^	118.33 ± 33.26 ^c^	139.14 ± 29.53 ^a^	141.33 ± 19.75 ^bc^
Total proteins	g/L	62.00 ± 5.32 ^a^	63.00 ± 1.87 ^abcd^	68.28 ± 3.98 ^def^	67.11 ± 4.82 ^ce^	64.76 ± 5.28 ^bf^
**Renal function**						
LDH	U/L	327.33 ± 26.00 ^ac^	693.00 ± 78.23 ^b^	351.88 ± 91.34 ^ac^	569.00 ± 89.50 ^a^	426.26 ± 49.25 ^c^
Urea	g/L	0.22 ± 0.04 ^ac^	0.62 ± 0.12 ^b^	0.43 ± 0.09 ^c^	0.49 ± 0.19 ^ab^	0.28 ± 0.05 ^a^
Creatinine	mg/L	4.61 ± 0.28 ^abcd^	7.70 ± 1.49 ^a^	4.18 ± 0.22 ^b^	4.95 ± 0.35 ^c^	4.68 ± 0.32 ^d^
**Lipid profile**						
Cholesterol	g/L	0.44 ± 0.16 ^ac^	1.09 ± 0.20 ^b^	0.46 ± 0.17 ^ad^	0.66 ± 0.19 ^c^	0.63 ± 0.12 ^cd^
TG	g/L	0.58 ± 0.09 ^acde^	4.51 ± 0.26 ^b^	0.41 ± 0.16 ^c^	0.72 ± 0.21 ^d^	1.01 ± 0.22 ^e^
HDL	g/L	0.22 ± 0.04 ^a^	0.14 ± 0.04 ^b^	0.21 ± 0.05 ^a^	0.17 ± 0.05 ^a^	0.17 ± 0.01 ^a^
LDL	g/L	0.20 ± 0.03 ^a^	0.26 ± 0.05 ^ab^	0.21 ± 0.09 ^ab^	0.21 ± 0.08 ^b^	0.21 ± 0.12 ^a^
AI	ratio	0.91 ± 0.05 ^a^	2.36 ± 0.03 ^d^	0.99 ± 0.05 ^b^	1.23 ± 0.04 ^c^	1.23 ± 0.10 ^bc^
**Minerals**						
Sodium	mmol/L	141.28 ± 1.60 ^a^	134.66 ± 9.71 ^ab^	141.14 ± 2.11 ^ab^	140.14 ± 1.57 ^b^	139.80 ± 2.48 ^ab^
Potassium	mmol/L	5.42 ± 0.54 ^ac^	4.41 ± 0.60 ^bc^	5.4 ± 1.15 ^ab^	5.39 ± 0.35 ^abc^	6.31 ± 1.33 ^c^
Chlore	mmol/L	106.71 ± 2.62 ^a^	97.00 ± 7.93 ^ab^	103.85 ± 1.95 ^ac^	101.42 ± 1.98 ^b^	98.60 ± 3.78 ^bc^

Data are reported to mean (*n* = 8) ± SD. Values in the same row and category not sharing a common letter (^a–d^) differs significantly at *p* < 0.05.

**Table 4:** Levels of antioxidant enzymes and oxidative stress markers in liver and kidney.

	MDA	T-SOD	Mn-SOD	CuZn-SOD	CAT	GSH	GPx
**Liver**							
NC	0.42 ± 0.05 ^a^	7.22 ± 1.57 ^a^	1.66 ± 0.44 ^a^	3.58 ± 0.24 ^ab^	5.78 ± 1.44 ^a^	2.36 ± 0.25 ^a^	3.22 ± 0.27 ^ac^
DC	6.37 ± 0.60 ^d^	16.21 ± 2.86 ^c^	3.64 ± 0.63 ^d^	11.80 ± 2.06 ^c^	18.42 ± 1.81 ^c^	4.38 ± 0.99 ^c^	7.20 ± 0.42 ^d^
Met	0.55 ± 0.06 ^b^	7.25 ± 2.46 ^a^	1.96 ± 0.46 ^c^	4.33 ± 1.30 ^b^	7.17 ± 0.77 ^a^	3.50 ± 0.97 ^b^	2.05 ± 0.71 ^a^
Am	1.07 ± 0.17 ^c^	8.30 ± 0.48 ^abc^	2.72 ± 0.54 ^b^	3.60 ± 1.34 ^a^	8.57 ± 1.71 ^b^	3.08 ± 0.35 ^abc^	2.75 ± 0.23 ^b^
F11	2.13 ± 0.98 ^abc^	14.52 ± 3.00 ^b^	2.93 ± 0.75 ^abc^	9.25 ± 0.50 ^c^	14.74 ± 3.88 ^abc^	3.38 ± 1.60 ^abc^	5.91 ± 0.94 ^cd^
**Kidney**							
NC	0.182 ± 0.02 ^ab^	8.30 ± 0.65 ^ab^	2.64 ± 0.38 ^a^	4.32 ± 0.98 ^a^	8.57 ± 0.96 ^a^	1.46 ± 0.18 ^a^	3.94 ±0.68 ^ae^
DC	0.92 ± 0.05 ^e^	12.63 ± 1.02 ^c^	4.01 ± 0.12 ^b^	9.99 ± 1.07 ^b^	12.86 ± 0.81 ^c^	2.13 ± 0.31 ^d^	7.53 ± 1.41 ^e^
Met	0.42 ± 0.03 ^cd^	10.04 ± 0.63 ^a^	2.79 ± 0.36 ^a^	5.39 ± 0.87 ^a^	10.32 ± 0.72 ^b^	1.48 ± 0.20 ^ab^	5.34 ± 1.26 ^bde^
Am	0.232 ± 0.09 ^ac^	7.93 ± 0.95 ^ab^	2.83 ± 0.59 ^a^	5.61 ± 0.26 ^a^	7.99 ± 1.69 ^ab^	1.14 ± 0.11 ^bc^	6.52 ± 1.18 ^cde^
F11	0.453 ± 0.09 ^bd^	7.16 ± 0.85 ^b^	3.44 ± 0.72 ^a^	4.75 ± 0.71 ^a^	7.82 ± 1.24 ^a^	1.22 ± 0.18 ^c^	5.45 ± 0.57 ^d^

Data are reported to mean (*n* = 3) ± SD. Values in the same row and category not sharing a common letter (^a–e^) differs significantly at *p* < 0.05. CAT, Mn-SOD, CuZn-SOD and GPx are expressed in in μmol/min/mg protein, GSH in μmol GSH/mg protein, MDA in nmol MDA/mg protein.

**Table 5:** Behavioral analysis using OFT and EPM.

	NC	DC	Met	Am	F11
**Open Field test**					
Total squares entries	42.32 ± 5.32 ^c^	12.21 ± 2.11 ^a^	29.21 ± 3.21 ^b^	25.43 ± 2.42 ^b^	27.43 ± 5.32 ^b^
Central squares entries	19.45 ± 3.23 ^d^	2.12 ± 0.98 ^a^	9.43 ± 1.21 ^b^	5.32 ± 1.05 ^ab^	4.98 ± 1.43 ^ab^
Time spent in central squares (%)	45.95 ± 4.23 ^c^	17.36 ± 3.32 ^a^	32.28 ± 2.79 ^b^	20.92 ± 2.56 ^a^	18.15 ± 3.26 ^a^
**Elevated Plus Maze test**					
Arm entries	35.60 ± 4.56 ^d^	3.21 ± 0.45 ^a^	29.32 ± 2.22 ^d^	7.75 ± 1.45 ^b^	12.25 ± 1.67 ^c^
Open arms entries	23.12 ± 3.21 ^d^	2.50 ± 0.32 ^a^	21.32 ± 3.17 ^c^	4.75 ± 0.89 ^a^	7.75 ± 1.22 ^b^
Time spent in open arms (%)	26.29 ± 5.43 ^d^	3.34 ± 1.12 ^a^	16.78 ± 5.69 ^c^	5.88 ± 3.45 ^a^	9.43 ± 4.01 ^b^

Data are reported to mean (*n* = 8) ± SD. Values in the same column and category not sharing a common letter (^a–d^) differs significantly at *p* < 0.05.

## Abbreviations

AD	Alzheimer disease
AI	Atherosclerosis index
AST	Aspartate aminotransferase
ALT	Alanine aminotransferase
FBG	Fasting blood glucose
AUC	Areas under curve
CAT	Catalase
DNS	Dinitrosalicylic acid
EPM	Elevated plus maze
GPx	Glutathione Peroxidase
GSH	Glutathione
HDL	High density lipoprotein
HGB	Hemoglobin
LDH	Lactate dehydrogenase
LDL	Low density lipoprotein
MDA	Malondialdehyde
*p*-NPG	*p*-Nitrophenyl-α-D-glucopyranoside
OFR	Overnight fasted rats
OFT	Open field test
OGTT	Oral glucose tolerance test
OS	Oxidative stress
PBS	Phosphate buffer solution
PPYG	Proteose-peptone yeast Glucose defined medium
RBC	Red blood corpuscles count
ROS	Radical oxygenated species
SOD	Superoxide dismutase
STZ-NA	Streptozotocine nicotinamide
TG	Triacylglycerols
HDL	High density lipoprotein
T2DM	Type 2 diabetes mellitus
WBC	White blood corpuscles count

## References

[1] Rains J.L., Jain S.K. (2011). Oxidative stress, insulin signaling, and diabetes. Free Radic. Biol. Med..

[2] Robertson R.P. (2004). Chronic oxidative stress as a central mechanism for glucose toxicity in pancreatic islet beta cells in diabetes. J. Biol. Chem..

[3] Wold L.E., Asli F.C., Jun R. (2005). Oxidative stress and stress signaling: Menace of diabetic cardiomyopathy. Acta Pharmacol. Sin..

[4] Evans J.L., Goldfine I.D., Maddux B.A., Grodsky G.M. (2003). Are Oxidative Stress—Activated Signaling Pathways Mediators of Insulin Resistance and β-Cell Dysfunction?. Diabetes.

[5] Henriksen J.E., Diamond-Stanic M.K., Marchionne E.M. (2011). Oxidative stress and the etiology of insulin resistance and type 2 diabetes. Free Radic. Biol. Med..

[6] Houstis N., Rosen E.D., Lander E.S. (2006). Reactive oxygen species have a causal role in multiple forms of insulin resistance. Nature.

[7] Ceriello A. (2003). New Insights on Oxidative Stress and Diabetic Complications May Lead to a “Causal” Antioxidant Therapy. Diabetes Care.

[8] Truswell A.S. (2002). Cereal grains and coronary heart disease. Eur. J. Clin. Nutr..

[9] Marventano S., Vetrani C., Vitale M., Godos J., Riccardi G., Grosso G. (2017). Whole Grain Intake and Glycaemic Control in Healthy Subjects: A Systematic Review and Meta-Analysis of Randomized Controlled Trials. Nutrients..

[10] Marmouzi I., Saidi N., Meddah B., Bouksaim M., Gharby S., El Karbane M., Serragui S., Cherrah Y., Faouzi M.E.A. (2016). Nutritional characteristics, biochemical composition and antioxidant activities of Moroccan Oat varieties. Food Meas..

[11] Rahimi R., Nikfar S., Larijani B., Abdollahi M. (2005). A review on the role of antioxidants in the management of diabetes and its complications. Biomed. Pharmacother..

[12] Shen R.L., Cai F.L., Dong J.L., Hu X.Z. (2011). Hypoglycemic Effects and Biochemical Mechanisms of Oat Products on Streptozotocin-Induced Diabetic Mice. J. Agric. Food Chem..

[13] Zhao Q., Hu X., Guo Q., Cui S.W., Xian Y., You S., Chen X., Xu C., Gao X. (2014). Physicochemical properties and regulatory effects on db/db diabetic mice of β-glucans extracted from oat, wheat and barley. Food Hydrocoll..

[14] Detka J., Kurek A., Basta-Kaim A., Kubera M., Lasoń W., Budziszewska B. (2013). Neuroendocrine link between stress, depression and diabetes. Pharmacol. Rep..

[15] Butterfield D.A., Domenico F.D., Barone E. (2014). Elevated risk of type 2 diabetes for development of Alzheimer disease: A key role for oxidative stress in brain. Biochim. Biophys. Acta.

[16] Rocher A., Colilla F., Ortiz M.L., Mendez E. (1992). Identification of the three major coeliac immunoreactive proteins and one α-amylase inhibitor from oat endosperm. FEBS Lett..

[17] Bischoff B.A.G.H. (1994). Pharmacology of α-glucosidase inhibition. Eur. J. Clin. Investig..

[18] Dong J., Cai F., Shen R., Liu Y. (2011). Hypoglycaemic effects and inhibitory effect on intestinal disaccharidases of oat beta-glucan in streptozotocin-induced diabetic mice. Food Chem..

[19] Marmouzi I., El Madani N., Charrouf Z., Cherrah Y., Faouzi M.E.A. (2015). Proximate analysis, fatty acids and mineral composition of processed Moroccan *Chenopodium quinoa Willd* and antioxidant properties according to the polarity. Phytothérapie.

[20] Saidi N., Saidi S., Hilali A., Benchekroun M., Al Faiz C., Bouksaim M., Shaimi N., Souihka A., Idrissi S.A., Gaboune F. (2013). Improvement of oat hexaploid lines’s groat nutritive value via hybridisation with tetraploid oat *A. magna*. Am. J. Res. Commun..

[21] Mori K., Kashiwagi A., Yomo T. (2011). Single-Cell Isolation and Cloning of *Tetrahymena thermophila* Cells with a Fluorescence-Activated Cell Sorter. J. Eukaryot. Microbiol..

[22] Lizard G., Gueldry S., Deckert V., Gambert P., Lagrost L. (1997). Evaluation of the cytotoxic effects of some oxysterols and of cholesterol on endothelial cell growth: Methodological aspects. Pathol. Biol..

[23] Lowry O.H., Rosebrough H.N., Farr A.L., Randall R.J. (1951). Protein measurement with the folin phenol reagent. J. Biol. Chem..

[24] Beauchamp C., Fridovich I. (1971). Superoxide dismutase: Improved assays and an assay applicable to acrylamide gels. Anal. Biochem..

[25] Mannervik B. (1985). Glutathione peroxidase. Methods Enzymol..

[26] Furman B.L. (2015). Streptozotocin-induced diabetic models in mice and rats. Curr. Protoc. Pharmacol..

[27] Kee K.T., Koh M., Oong L.X., Ng K. (2013). Screening culinary herbs for antioxidant and α-glucosidase inhibitory activities. Int. J. Food Sci. Technol..

[28] Walf A.A., Frye C.A. (2007). The use of the elevated plus maze as an assay of anxiety-related behavior in rodents. Nat. Protoc..

[29] Prut L., Belzung C. (2003). The open field as a paradigm to measure the effects of drugs on anxiety-like behaviors: A review. Eur. J. Pharmacol..

[30] Aebi H. (1984). Catalase in vitro. Methods Enzymol..

[31] Moron M.S., Depierre J.W., Mannervik B. (1979). Levels of glutathione, glutathione reductase and glutathione S-transferase activities in rat lung and liver. Biochim. Biophys. Acta.

[32] Ohkawa H., Ohishi N., Yagi K. (1979). Assay of lipid peroxides in animal tissue by thio barbituric acid reaction. Anal. Biochem..

[33] Efferth T., Egon K. (2011). Complex interactions between phytochemicals. The multi-target therapeutic concept of phytotherapy. Curr. Drug Targets.

[34] Lin Y., Sun Z. (2010). Current views on type 2 diabetes. J. Endocrinol..

[35] Lebovitz H.E. (1997). Alpha-Glucosidase Inhibitors. Endocrinol. Metab. Clin. N. Am..

[36] Van de Laar F.A., Lucassen P.L.B.J., Akkermans R.P., Van de Lisdonk E.H., Rutten G.E.H.M., Van Weel C. (2005). Alpha-glucosidase inhibitors for type 2 diabetes mellitus. Cochrane Database Syst. Rev..

[37] Krause H.P., Ahr H.J. (1996). Pharmacokinetics and Metabolism of Glucosidase Inhibitors. Oral Antidiabetics.

[38] Balfour J.A., McTavish D. (1993). Acarbose: An Update of its Pharmacology and Therapeutic Use in Diabetes Mellitus. Drugs.

[39] Wang P.Y., Kaneko T., Wang Y., Sato A. (1999). Acarbose alone or in combination with ethanol potentiates the hepatotoxicity of carbon tetrachloride and acetaminophen in rats. J. Hepatol..

[40] Evert A.B., Boucher J.L., Cypress M., Dunbar S.A., Franz M.J., Mayer-Davis E.J., Neumiller J.J., Nwankwo R., Verdi C.L., Urbanski P. (2014). Nutrition Therapy Recommendations for the Management of Adults With Diabetes. Diabetes Care.

[41] Thu Phan M.A., Wang J., Tang J., Lee Y.Z., Ng K. (2013). Evaluation of α-glucosidase inhibition potential of some flavonoids from Epimedium brevicornum. LWT Food Sci. Technol..

[42] Ramakrishna R., Sarkar D., Schwarz P., Shetty K. (2017). Phenolic linked anti-hyperglycemic bioactives of barley (*Hordeum vulgare* L.) cultivars as nutraceuticals targeting type 2 diabetes. Ind. Crops Prod..

[43] Malunga L.N., Eck P. (2016). Inhibition of intestinal α-glucosidase and glucose absorption by feruloylated arabinoxylan mono-and oligosaccharides from corn bran and wheat aleurone. J. Nutr. Metab..

[44] Hemalatha P., Bomzan D.P., Rao B.S., Sreerama Y.N. (2016). Distribution of phenolic antioxidants in whole and milled fractions of quinoa and their inhibitory effects on α-amylase and α-glucosidase activities. Food Chem..

[45] Daou C., Zhang H. (2012). Oat Beta-Glucan: Its Role in Health Promotion and Prevention of Diseases. Compr. Rev. Food Sci. Food Saf..

[46] Shen X.L., Zhao T., Zhou Y., Shi X., Zou Y., Zhao G. (2016). Effect of oat β-glucan intake on glycaemic control and insulin sensitivity of diabetic patients: A meta-analysis of randomized controlled trials. Nutrients.

[47] Wanga Q., Ellisa P.R. (2014). Oat β-glucan: Physico-chemical characteristics in relation to its blood-glucose and cholesterol-lowering properties. Br. J. Nutr..

[48] Radenković M., Stojanović M. (2016). Milica Prostran Experimental diabetes induced by alloxan and streptozotocin: The current state of the art. J. Pharmacol. Toxicol. Methods.

[49] Masiello P., Broca C., Gross R., Roye M., Manteghetti M., Hillaire-Buys D., Novelli M., Ribes G. (1998). Experimental NIDDM: Development of a New Model in Adult Rats Administered Streptozotocin and Nicotinamide. Diabetes.

[50] Swanston-Flat S.K., Day C., Bailey C.J., Flatt P.R. (1990). Traditional plant treatment for diabetes: Studies in normal and streptozotocin diabetic mice. Diabetologia.

[51] American Diabetes Association (2015). 2. Classification and Diagnosis of Diabetes. Diabetes Care.

[52] Taylor F., Huffman M.D., Macedo A.F., Moore T.H.M., Burke M., Davey Smith G., Ward K., Ebrahim S. (2013). Statins for the primary prevention of cardiovascular disease. Cochrane Database Syst. Rev..

[53] Ohaeri O.C. (2001). Effect of garlic oil on the levels of various enzymes in the serum and tissue of streptozotocin diabetic rats. Biosci. Rep..

[54] Palsamy P., Subramanian S. (2008). Resveratrol, a natural phytoalexin, normalizes hyperglycemia in streptozotocin-nicotinamide induced experimental diabetic rats. Biomed. Pharmacother..

[55] Natali A., Ferrannini E. (2006). Effects of metformin and thiazolidinediones on suppression of hepatic glucose production and stimulation of glucose uptake in type 2 diabetes: A systematic review. Diabetologia.

[56] King G.L., Loeken M.R. (2004). Hyperglycemia-induced oxidative stress in diabetic complications. Histochem. Cell. Biol..

[57] Mahadev K., Zilbering A., Zhu L., Goldstein B.J. (2001). Insulin-stimulated Hydrogen Peroxide Reversibly Inhibits Protein-tyrosine Phosphatase 1B in Vivo and Enhances the Early Insulin Action Cascade. J. Biol. Chem..

[58] Porokhovnik L.N., Passekov V.P., Gorbachevskaya N.L., Sorokin A.B., Veiko N.N., Lyapunova N.A. (2015). Active ribosomal genes, translational homeostasis and oxidative stress in the pathogenesis of schizophrenia and autism. Psychiatr. Genet..

[59] Genet S., Kale R.K., Baquer N.Z. (2002). Alterations in antioxidant enzymes and oxidative damage in experimental diabetic rat tissues: Effect of vanadate and fenugreek (*Trigonella foenum graecum*). Mol. Cell. Biochem..

[60] Rajendran P., Nandakumar N., Rengarajan T., Palaniswami R., Gnanadhas E.N., Lakshminarasaiah U., Gopas J., Nishigaki I. (2014). Antioxidants and human diseases. Clin. Chim. Acta.

[61] Godin D.V., Wohaieb S.A., Garnett M.E., Goumeniouk A.D. (1988). Antioxidant enzyme alterations in experimental and clinical diabetes. Mol. Cell. Biochem..

[62] Peterson D.M. (2001). Oat Antioxidants. J. Cereal Sci..

[63] Chen C.Y., Milbury P.E., Kwak H.K., Collins F.W., Samuel P., Blumberg J.B. (2004). Avenanthramides and Phenolic Acids from Oats Are Bioavailable and Act Synergistically with Vitamin C to Enhance Hamster and Human LDL Resistance to Oxidation. J. Nutr..

[64] Wang P., Chen H., Zhu Y., McBride J., Fu J., Sang S. (2015). Oat Avenanthramide-C (2c) Is Biotransformed by Mice and the Human Microbiota into Bioactive Metabolites. J. Nutr..

[65] Salkovic-Petrisic M., Knezovic A., Hoyer S., Riederer P. (2013). What have we learned from the streptozotocin-induced animal model of sporadic Alzheimer’s disease, about the therapeutic strategies in Alzheimer’s research. J. Neural Transm..

[66] Samy D.M., Ismail C.A., Nassr R.A., Zeitoun T.M., Nomair A.M. (2016). Down stream modulation of extrinsic apoptotic pathway in streptozotocin-induced Alzheimer's dementia in rats: Erythropoietin versus curcumin. Eur. J. Pharmacol..

[67] Rebolledo-Solleiro D., Crespo-Ramírez M., Roldán-Roldán G., Hiriart M., Pérez de la Mora M. (2013). Role of thirst and visual barriers in the differential behavior displayed by streptozotocin-treated rats in the elevated plus-maze and the open field test. Physiol. Behav..

[68] Garabadu D., Krishnamurthy S. (2014). Diazepam Potentiates the Antidiabetic, Antistress and Anxiolytic Activities of Metformin in Type-2 Diabetes Mellitus with Cooccurring Stress in Experimental Animals. BioMed Res. Int..

